# Trends in Mortality Among Adults With Acute Myocardial Infarction With Cardiogenic Shock in the United States, 1999-2023

**DOI:** 10.1016/j.jscai.2025.103711

**Published:** 2025-06-12

**Authors:** Abdullah Naveed Muhammad, Sivaram Neppala, Himaja Dutt Chigurupati, Bazil Azeem, Rabia Iqbal, Muhammad Omer Rehan, Priya Hotwani, Ahila Ali, Sowjanya Kapaganti, Mushood Ahmed, Mobeen Zaka Haider, Yasar Sattar, Jamal S. Rana, Sourbha Dani

**Affiliations:** a Department of Cardiology, Dow Medical College, Dow University of Health Sciences, Karachi, Pakistan; b Division of Cardiology, The University of Texas Health Sciences Center, San Antonio, Texas; c Department of Internal Medicine, New York Medical College at Saint Michael’s Medical Center, Newark, New Jersey; d Department of Internal Medicine, Parkview Health, Fort Wayne, Indiana; e Division of Cardiology, Creighton University, Omaha, Nebraska; f Department of Medicine, Rawalpindi Medical University, Rawalpindi, Pakistan; g Department of Cardiology, West Virginia University, Morgantown, West Virginia; h Department of Cardiology, The Permanente Medical Group, Oakland, California; i Department of Cardiology, Lahey Hospital and Medical Center, Burlington, Massachusetts

**Keywords:** acute myocardial infarction, cardiogenic shock, mortality, race, sex

## Abstract

**Background:**

Cardiogenic shock (CS) elevates mortality rates among patients with acute myocardial infarction (AMI), yet there are insufficient data on trends in mortality. This study seeks to elucidate demographic patterns and mortality statistics.

**Methods:**

We analyzed data from the Centers for Disease Control and Prevention's Wide-ranging ONline Data for Epidemiologic Research (1999-2023) to evaluate mortality related to CS among AMI patients aged 25 years and above. Age-adjusted mortality rates (AAMR) per 100,000 patients and average annual percentage changes were calculated using JoinPoint regression analysis to explore mortality trends.

**Results:**

Cardiogenic shock contributed to 187,838 deaths among AMI patients aged 25 years and older. Between 1999 and 2023, the AAMR fell from 5.4 to 3.3 per 100,000, reflecting an average annual percentage change of –2.02. The most significant reduction occurred from 1999 to 2011, followed by a notable increase from 2011 to 2021 (annual percent change, 3.32). Disparities are apparent, as men have higher AAMR than women (4.5 vs 2.5), with Hispanic individuals at the most significant risk (AAMR, 3.5), followed by Whites (AAMR, 3.4). Regionally, West Virginia has the highest AAMR at 5.3, whereas Minnesota has the lowest at 2.3. Additionally, rural areas report higher AAMR than urban ones (4.0 vs 3.2).

**Conclusions:**

The recent increase in mortality rates between 2011 and 2021 due to CS in AMI and disparities among men, Hispanic individuals, and people living in rural areas—calls for urgent attention. By applying focused interventions and improving health care access, we can bridge these gaps and enhance patient outcomes.

## Introduction

Cardiogenic shock (CS) is an alarming complication of acute myocardial infarction (AMI) that continues to play a role in high-mortality rates, even with the advent of advanced therapeutic interventions.^1^^,^^2^ This condition occurs when the heart cannot pump enough blood to meet the body's requirements, resulting in profound end-organ hypoperfusion and hypoxia. It plays a pivotal role in the mortality rates of hospitalized patients, affecting nearly 10% of those admitted with AMI.^3^ Alarmingly, despite a decline in the incidence of AMI, mortality rates have remained stable, with 40% to 45% of these patients succumbing to death within just 30 days.^4^, ^5^, ^6^

The management of CS typically involves vasopressors and inotropes, but their efficacy can be questionable and may increase myocardial oxygen demand, worsening tissue perfusion.^2^ The Should We Emergently Revascularize Occluded Coronaries for Cardiogenic Shock trial showed that early revascularization significantly improves survival rates by 6 months.^7^^,^^8^ The CULPRIT-SHOCK trial found that treating only the culprit lesion reduces 30-day mortality by 8.2% compared to multivessel percutaneous coronary intervention.^9^ Although innovations like extracorporeal membrane oxygenation and ventricular assist device have emerged, trials show limited clinical benefits and potential complications associated with ECMO in AMI cases.^10^, ^11^, ^12^, ^13^, ^14^ The DanGer Shock study found that microaxial flow pumps decrease all-cause mortality at 180 days compared to standard care, despite higher adverse events.^15^

Moreover, research highlights disparities in the incidence and outcomes of CS, particularly among older adults suffering from delayed recognition and nonrevascularization.^16^, ^17^, ^18^ The increasing prevalence of cardiovascular risk factors in younger populations further underscores the need to assess mortality trends.^11^ Socioeconomic status and health care access inequalities also contribute to adverse outcomes, especially in rural areas.^19^ This study utilizes the Centers for Disease Control and Prevention's Wide-ranging ONline Data for Epidemiologic Research (CDC WONDER) database to analyze mortality trends in CS among AMI patients aged 25 years and older in the United States (US), emphasizing the need for targeted interventions.

## Methods

### Study design

The data for this study were derived from the CDC WONDER database, a highly respected and comprehensive repository of death certificates.^20^ Covering a significant period from 1999 to 2023, this meticulously curated data set was explicitly designed to investigate deaths linked to CS among individuals with AMI, utilizing precise ICD-10 codes to uphold data integrity.^21^ This extensive collection features death certificates from all 50 states and the District of Columbia, making it an invaluable resource for analyzing mortality trends related to various diseases in adults aged 25 years and older.

Additionally, this study relied on deidentified data sets that are publicly available and produced by governmental organizations. Due to the nature of these data, institutional review board approval was optional. Nonetheless, the research rigorously adhered to the Strengthening the Reporting of Observational Studies in Epidemiology (STROBE) guidelines, ensuring the high standard of scientific integrity and reliability of our findings.

### Study cohort

This study focused on adults aged 25 years and older diagnosed with CS among AMI patients between 1999 and 2023. We rigorously analyzed death records from the Multiple Causes of Death Public Use registry to identify cases of mortality linked to AMI within this specific population. By employing the International Classification of Diseases (ICD) codes, we specifically targeted instances of CS (R570) and AMI (I21.0, I21.1, I21.2, I21.3, I21.9, I22.0, I22.1, I22.8, and I22.9), ensuring a precise and thorough examination of these critical health outcomes.

### Data extraction

This study meticulously developed a comprehensive data set encompassing essential mortality-related factors, including population size, year of death, geographic location, demographic characteristics, urban-rural classification, regional distribution, and state-specific categories. The demographic data include vital variables such as age and race/ethnicity. Locations of death are categorized across various health care settings, including outpatient facilities, emergency rooms, and inpatient hospitals, while also distinguishing cases of death occurring on arrival or with unknown status, as well as deaths at home, in hospice, nursing homes, or long-term care facilities. Race and ethnicity classifications are divided into Hispanic, non-Hispanic (NH) White, NH Black, and NH other. These carefully collected parameters have undergone validation through the CDC WONDER database analysis, facilitating a nuanced understanding of mortality trends and their implications.

To ensure accurate population assessment, we employed the National Center for Health Statistics Urban-Rural Classification Scheme, which classifies counties as urban (including large central metropolitan, large fringe metropolitan, medium metropolitan, and small metropolitan) or nonmetropolitan (comprising micropolitan and noncore areas) based on the 2013 US Census. Additionally, utilizing definitions from the US Census Bureau, regions were delineated into the Northeast, Midwest, South, and West, enhancing our findings' specificity and relevance. Through these robust classifications, our study provides critical insights into mortality patterns across diverse populations.

### Statistical analysis

The study comprehensively analyzed the crude mortality rate and age-adjusted mortality rate (AAMR) per 100,000 individuals to examine nationwide mortality trends thoroughly. This involved meticulously calculating the total fatalities associated with CS within the patient population for each year under consideration. Following established research methodologies, the AAMR was computed by standardizing these CS-related deaths against the 2000 US population while generating 95% CIs to ensure accuracy. To determine the annual percent change (APC) and its corresponding 95% CI for AAMR, we utilized the JoinPoint Regression Program (Joinpoint V 4.9.0.0, National Cancer Institute). Utilizing AAMR enabled meaningful comparisons of mortality rates across diverse populations and historical periods. The study revealed significant mortality trends by analyzing these rates and highlighted notable fluctuations over time by employing log-linear regression models. Earlier studies employed AAMR, APC, and average annual percentage changes (AAPC) to identify trends and differences in mortality among various demographics.^22^^,^^23^

## Results

Between 1999 and 2023, CS among AMI patients accounted for a total of 187,838 deaths in adults aged 25 years and older in the US (Supplemental Table S1).

A total of 181,406 deaths (96.6%) occurred in medical facilities, whereas 2237 (1.2%) were reported in nursing homes/long-term care facilities, 1130 (0.6%) in hospice settings, and 1954 (1.0%) at home. Given the overwhelming majority of deaths occurring in hospitals, the inclusion of out-of-hospital deaths does not substantially alter the observed mortality trends. Although fatalities in nonhospital settings remain a small fraction of total mortality, these numbers are reported transparently in Supplemental Table S2 to provide complete clarity on the data set’s composition. An illustration summarizing the study's characteristics and findings is presented in the Central Illustration.

**Central Illustration:**
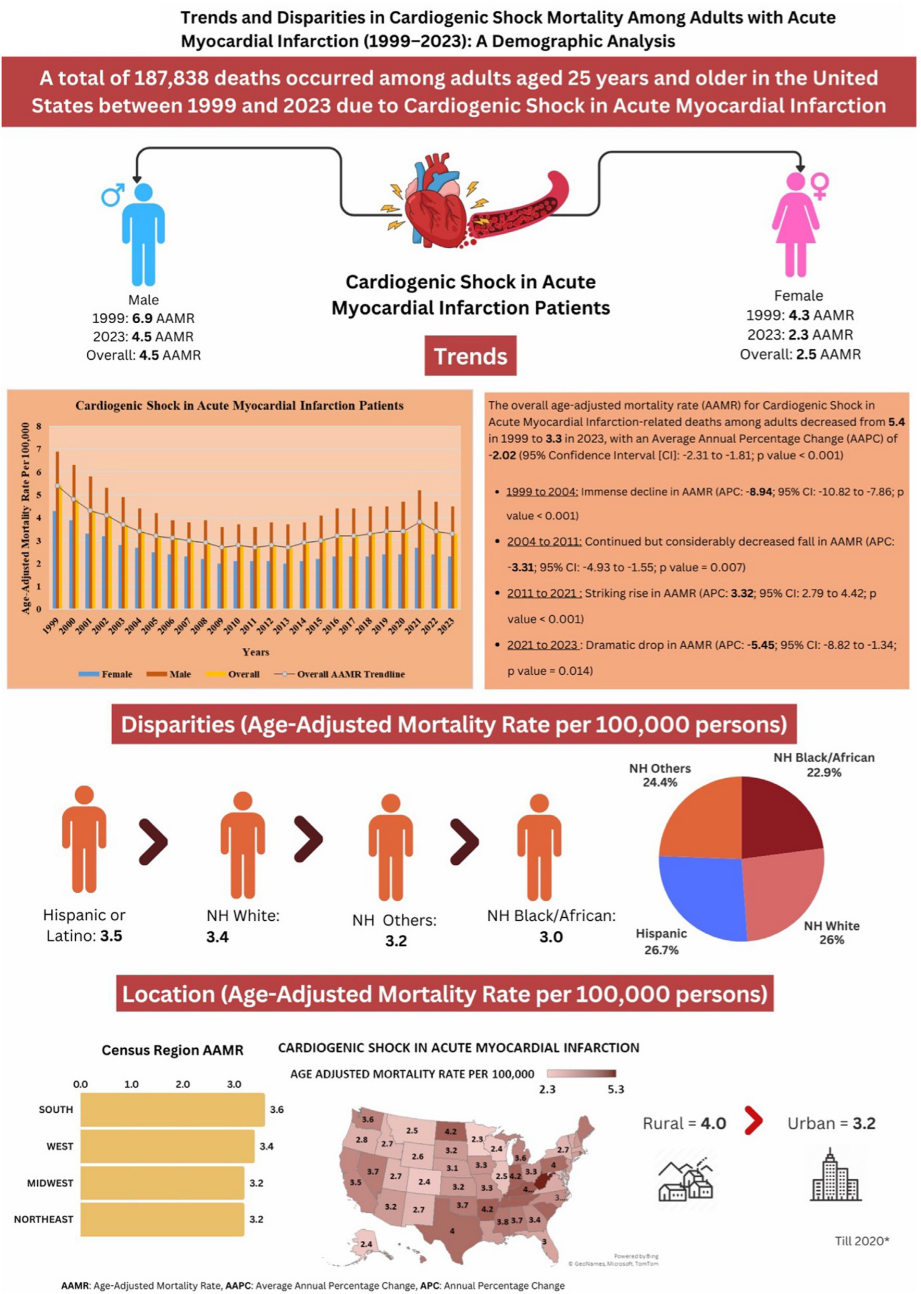
**Trends in demographics and disparities in cardiogenic shock–related mortality among adults with acute myocardial infarction in the United States from 1999 to 2023.** AAMR, age-adjusted mortality rate; AAPC, average annual percentage change; APC, annual percent change; NH, non-Hispanic.

### Annual trends for CS in AMI-related AAMR

The overall AAMR for CS among AMI-related deaths among adults decreased from 5.4 in 1999 to 3.3 in 2023, with an AAPC of –2.02 (95% CI, –2.31 to –1.81; *P* < .001). Notably, the AAMR showed an immense decline from 1999 to 2004 (APC, –8.94; 95% CI, –10.82 to –7.86; *P* < .001), followed by a continued but considerably decreased fall from 2004 to 2011 (APC, –3.31; 95% CI, –4.93 to –1.55; *P* = .007). This was followed by a notable increase from 2011 to 2021 (APC, 3.32; 95% CI, 2.79-4.42; *P* < .001), and finally, a significant decline from 2021 to 2023 (APC, –5.45; 95% CI, –8.82 to –1.34; *P* = .01) (Supplemental Table S3).

### CS in AMI-related AAMR stratified by sex

Throughout the study, men exhibited considerably higher AAMR than women (overall AAMR for men, 4.5; 95% CI, 4.4-4.6; AAMR for women, 2.5; 95% CI, 2.4-2.6). On average, the AAMR of both men and women decreased from 1999 to 2023, with the decrease more prominent in women (men: AAPC, –1.78; CI, –2.01 to –1.59; *P* < .001; women: AAPC, –2.57; 95% CI, –2.88 to –2.28; *P* < .001).

**Figure 1:**
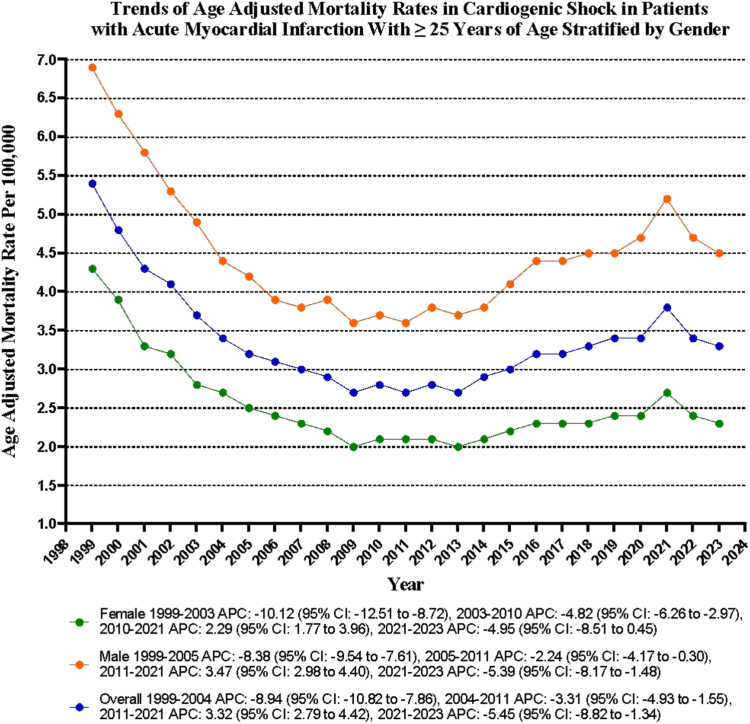
**Overall and sex-stratified cardiogenic shock–related age-adjusted mortality rates per 100,000 adults with acute myocardial infarction in the United States from 1999 to 2023.** APC, annual percent change.

The AAMR for men decreased exceptionally from 6.9 in 1999 to 4.2 in 2005 (APC, –8.38; 95% CI, –9.54 to –7.61; *P* < .001), followed by an appreciable fall to 3.6 by 2011 (APC, –2.24; 95% CI, –4.17 to –0.30; *P* = .02). It then exhibited a significant climb to 5.2 by 2021 (APC, 3.47; 95% CI, 2.98-4.40; *P* < .001) and, finally, a marked decline to 4.5 by 2023 (APC, –5.39; 95% CI, –8.17 to –1.48; *P* = .006). Similarly, the AAMR for women also initially decreased significantly from 4.3 in 1999 to 2.8 in 2003 (APC, –10.12; 95% CI, –12.51 to –8.72; *P* < .001), proceeded by a remarkable drop to 2.1 by 2010 (APC, –4.82; 95% CI, –6.26 to –2.97; *P* = .001). It then displayed a profound ascent to 2.7 by 2021 (APC, 2.29; 95% CI, 1.77-3.96; *P* = .009), followed by no significant change by 2023 (APC, –4.95; 95% CI, –8.51 to 0.45; *P* = .07) (Supplemental Table S4 and Figure 1).

### CS in AMI-related AAMR stratified by race/ethnicity

Slight variability in AAMR was found among different racial/ethnic groups. The AAMR were highest among Hispanics, followed by NH Whites, NH others, and NH Blacks (overall AAMR: Hispanic: 3.5; 95% CI, 3.2-3.8; NH White: 3.4; 95% CI, 3.3-3.5; NH other: 3.2; 95% CI, 2.9-3.6; NH Black: 3.0; 95% CI, 2.8-3.3).

**Figure 2:**
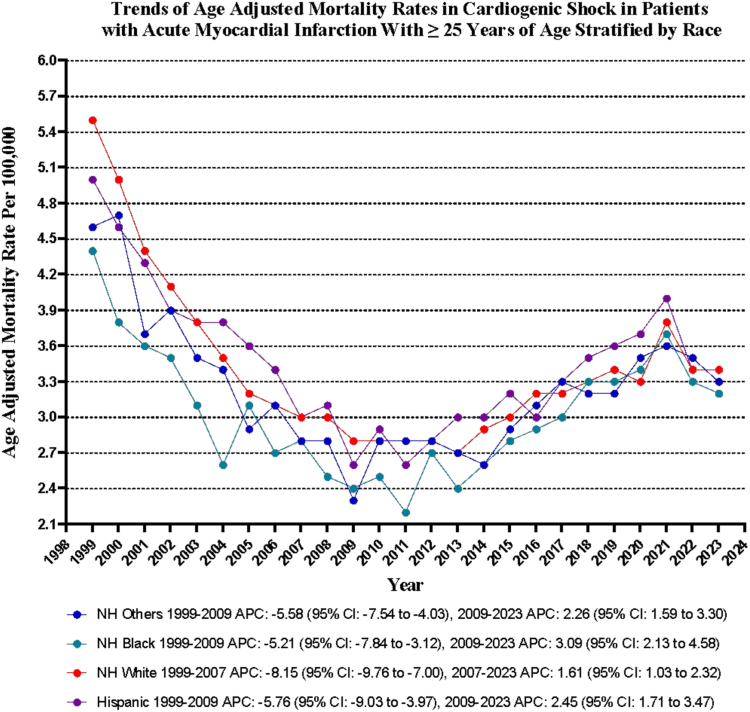
**Cardiogenic shock–related age-adjusted mortality rates per 100,000 stratified by race in adults with acute myocardial infarction in the United States, 1999 to 2023.** APC, annual percent change.

The AAMR of all the races decreased to variable degrees from 1999 to 2023 except for NH Blacks, with the decrease most pronounced in NH White (NH White: AAPC, –1.75; 95% CI, –2.05 to –1.48; *P* < .001; NH other: AAPC, –1.08; 95% CI, –1.43 to –0.61; *P* < .001; Hispanic: AAPC, –1.06; 95% CI, –1.59 to –0.45; *P* < .001; NH Black: AAPC, –0.45; 95% CI, –1.02 to 0.27; *P* = .20) (Supplemental Table S5 and Figure 2).

### CS in AMI-related AAMR stratified by geographical regions

Variations in AAMR were observed among different states, with AAMR ranging from as low as 2.3 (95% CI, 2.2-2.4) in Minnesota to the highest of 5.3 (95% CI, 5.0-5.5) in West Virginia. States falling within the top 90th percentile included Arkansas, Indiana, Kentucky, North Dakota, Rhode Island, Tennessee, and West Virginia, which had approximately twice higher AAMR compared to states in the lower 10th percentile, which included Alaska, Colorado, Illinois, Maryland, Minnesota, Montana, Wisconsin, and Wyoming (Supplemental Table S6).

**Figure 3:**
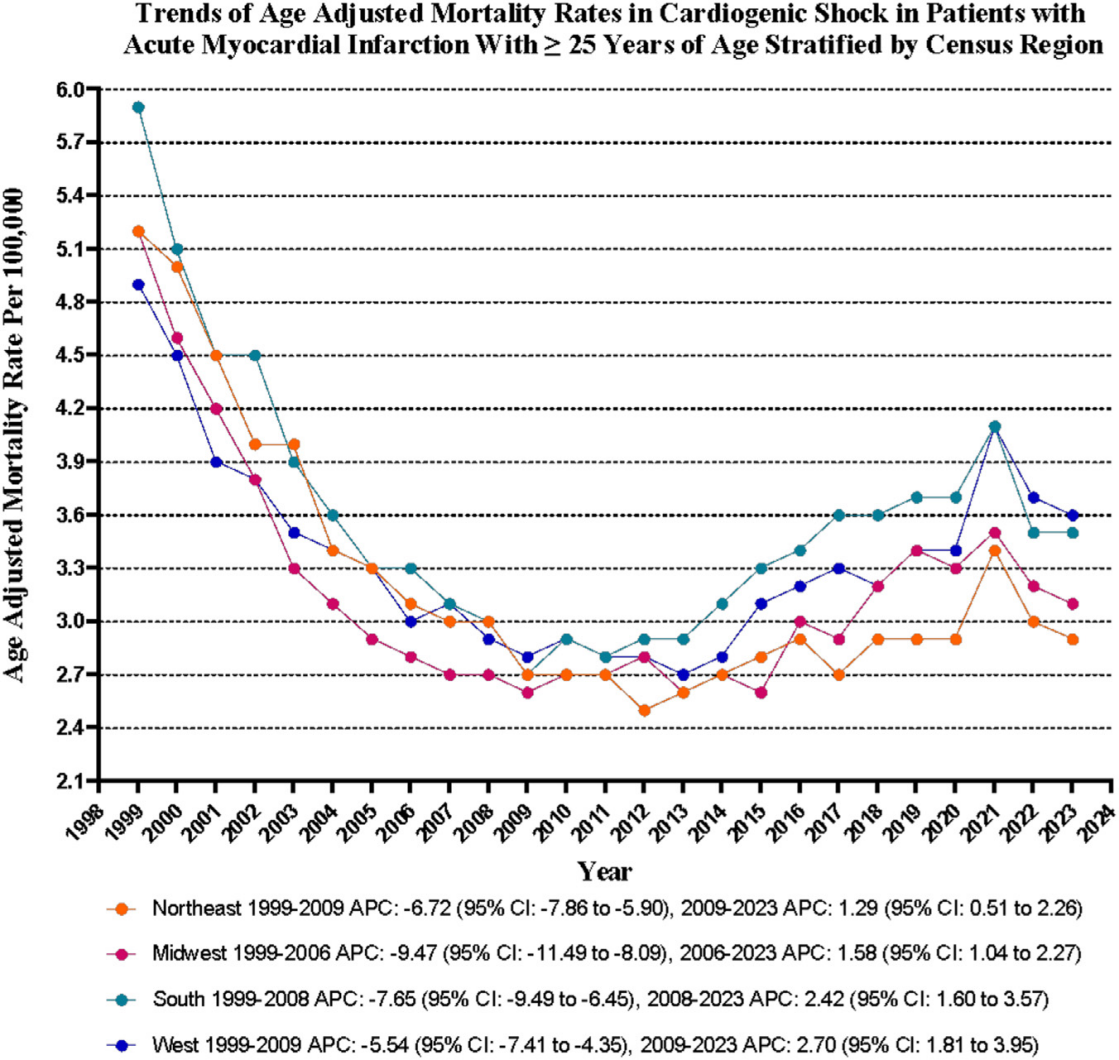
**Cardiogenic shock–related age-adjusted mortality rates per 100,000 stratified by regions in adults (≥25 years) with acute myocardial infarction in the United States, 1999 to 2023.** APC, annual percent change.

On average, over the study period, the highest mortality was observed in the Southern region (AAMR, 3.6; 95% CI, 3.5-3.7), followed by the Western (AAMR, 3.4; 95% CI, 3.2-3.5), Northeastern (AAMR, 3.2; 95% CI, 3.1-3.4), and Midwestern regions (AAMR, 3.2; 95% CI, 3.0-3.3) (Supplemental Table S7 and Figure 3).

**Figure 4:**
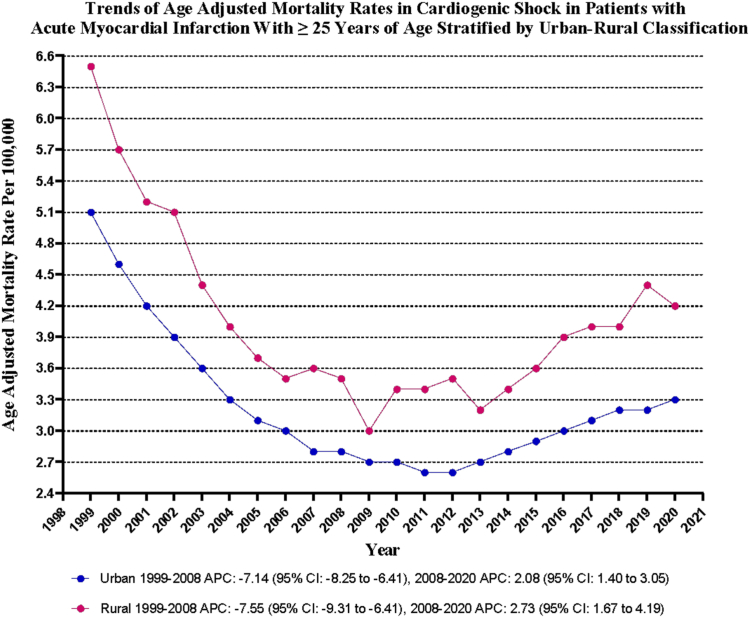
**Cardiogenic shock–related age-adjusted mortality rates per 100,000 stratified by urbanization in adults (≥25 years) with acute myocardial infarction in the United States, 1999 to 2023.** APC, annual percent change.

Rural areas exhibited relatively higher AAMR than urban areas throughout the study period, with overall AAMR of 4.0 (95% CI, 4.0-4.0) and 3.2 (95% CI, 3.1-3.2), respectively. The AAMR of both urban and rural areas decreased from 1999 to 2020, with the decrease slightly more pronounced in urban areas (urban: AAPC, –2.01; 95% CI, –2.34 to –1.71; *P* < .001; rural: AAPC, –1.79; 95% CI, –2.26 to –1.37; *P* < .001) (Supplemental Table S8 and Figure 4).

## Discussion

In this comprehensive analysis of mortality data from the Centers for Disease Control and Prevention in the US from 1999 to 2023, we identified the following critical findings regarding the substantial impact of mortality rates associated with CS in patients suffering from AMI: (1) The mortality rate of CS among adults aged 25 years and older with AMI was 187,838 deaths in the US from 1999 to 2023. (2) Despite decreasing mortality trends from 1999 to 2011, the AAMR rose significantly from 2011 to 2021, with an APC of 3.32, raising concern. (3) Men had higher AAMR than women, and both men and women saw a decrease from 1999 to 2023, with women showing a more significant decline. (4) Overall, AMMR for men and women dropped from 1999 to 2023, except for NH Blacks, which remained unchanged.

Our analysis provides compelling evidence of shifting trends in AAMR associated with CS within the context of AMI-related deaths over the past 2 decades (1999-2023). The remarkable decline observed from 1999 to 2004 (APC, –8.94) highlights the significant progress made in early revascularization techniques, advancements in critical care, and the implementation of standardized ST-elevation myocardial infarction protocols. Research indicates that early revascularization significantly reduces mortality rates in patients with CS, with some studies showing a concerning 40% to 50% mortality rate even with available treatments.^24^^,^^25^ The gradual decrease from 2004 to 2011 (APC, –3.31) reflects a stabilization phase as these effective treatment strategies became more widely adopted. However, the subsequent increase in mortality rates from 2011 to 2021 (APC, +3.32) may suggest the growing complexity of the AMI patient population and the challenges of integrating advanced treatment approaches, such as mechanical circulatory support devices like Impella and ECMO, with which complications and resource allocation issues have been encountered. Prior studies have revealed that hospitalizations for AMI have tripled in the US since 2008, particularly among young adults under the age of 65 years.^26^^,^^27^ Research indicates that the increasing mortality rates may be linked to heightened awareness of risk factors and the diagnosis of CS-AMI in patients admitted to the hospital with multiorgan dysfunction.^28^^,^^29^ Our study findings align with prior research, which indicated rising mortality trends in patients with CS-AMI since 2010.^30^^,^^31^

Earlier studies have reported an increase in cases of CS requiring mechanical ventilation among COVID-19 patients admitted for AMI.^32^^,^^33^ Our analysis supports these findings, showing a similar increase in mortality during the pandemic. The significant decrease in mortality from 2021 to 2023 (APC, –5.45) likely reflects improved patient selection, enhanced treatment methods, and necessary adjustments due to the COVID-19 pandemic. These observations emphasize the critical need for ongoing innovation in managing CS conditions.

We highlight critical sex-based differences in AAMR associated with CS in AMI cases. Notably, men consistently show higher AAMR than women (4.5% vs 2.5%), whereas the decline in mortality rates for women has been remarkably significant over the past 2 decades. These results align with previous studies that have indicated a higher mortality rate among men than among women.^31^ Encouragingly, women have shown an impressive decrease in AAMR, with an AAPC of –2.57, whereas men lag at –1.78. The most pronounced reduction for men occurred from 1999 to 2005 (APC, –8.38), but alarmingly, rates began to rise again from 2011 to 2021 (APC, +3.47). Women are often underrepresented in research on CS, making up less than one-third of the analyzed cases.^34^ They also receive fewer guideline-directed treatments, like mechanical circulatory support.^34^^,^^35^ The lower AAMR in women relative to men might indicate differences in how symptoms present, as women frequently show atypical symptoms that can result in underdiagnosis of AMI-CS.^36^ Despite the higher short-term mortality rates, long-term outcomes are comparable across sexes.^35^ These findings highlight the immediate necessity for better health care access and tailored treatment options for women to address disparities in treatment and outcomes.^37^

Additionally, the severity of CS in patients with AMI serves as a crucial determinant of clinical outcomes. It exhibits significant variability based on factors such as the extent of myocardial damage, intervention timing, and multiorgan dysfunction. Patients who present with more severe manifestations of CS often experience refractory hypotension, elevated lactate levels, and a requirement for mechanical circulatory support, all of which are correlated with increased mortality rates.^3^ However, administrative data sets, such as CDC WONDER, do not capture these detailed clinical variables, limiting the capacity to adjust for severity in mortality trend analyses. This underscores the necessity of integrating comprehensive clinical data for a more accurate interpretation of disparities in outcomes among diverse demographic subgroups.^2^^,^^9^^,^^10^

Our analysis revealed alarming racial and ethnic disparities, with Hispanic individuals experiencing the highest AAMR at 3.5, followed closely by NH Whites at 3.4. Despite the decline in AAMR over the past 2 decades, NH Blacks have witnessed the least improvement, underscoring ongoing inequities in health care access. Previous studies have shown that NH Black patients are less likely to receive critical interventions, such as intraaortic balloon pumps and percutaneous coronary interventions, compared to White patients.^38^ Furthermore, Hispanic patients exhibit lower rates of coronary angiography, which correlates with higher mortality rates.^39^ Socioeconomic factors play a significant role in these disparities, particularly impacting NH Black individuals.^40^^,^^41^ There is an urgent need for culturally tailored public health strategies to enhance outcomes for marginalized populations by improving access to preventive health care.^41^ Although disparities continue, advancements in health care delivery indicate that ongoing reforms and quality initiatives may help bridge these gaps in the future.^40^

The AAMR differed notably across the US, with Minnesota at 2.3 and West Virginia at 5.3. These statistics underscore significant disparities in health care outcomes. The Southern region exhibited the highest average AAMR at 3.6, whereas the Midwest and Northeast had lower rates of 3.2. Past studies using the National Inpatient Sample also indicated that Southern regions experienced higher mortality rates than others.^42^ Rural areas consistently exhibited higher AAMR of 4.0 compared to urban areas with a rate of 3.2. This trend persisted despite an overall decline in AAMR from 1999 to 2023. The reduction in AAMR was more significant in urban settings, highlighting ongoing challenges in health care delivery in rural regions. These findings emphasize the urgent need for targeted strategies to address disparities, particularly in high-mortality states and rural communities.^42^ Investments in health care access, infrastructure, and region-specific public health interventions are essential for improving health outcomes.^42^ To address these disparities, a multifaceted approach is necessary, considering both geographical and social determinants of health.

### Limitations

This study has limitations due to reliance on death certificate data, which may introduce misclassification bias. We used only ICD-10 codes from 1999 onward, provided by the CDC WONDER database. Although this approach ensures consistency, it does not distinguish between type I and type II NSTEMI or consider the potential overclassification of AMI-CS during the era of high-sensitivity troponin. The absence of clinical context, lab data, and procedural details in death certificates further hinders our ability to differentiate various causes of CS, such as those stemming from acute decompensated heart failure compared to primary AMI. Although the transition from ICD-9 to ICD-10 is irrelevant to our study, the inherent limitations of ICD coding systems remain pertinent.

Although our data set captures deaths occurring in both hospital and nonhospital environments, the overwhelming majority (96.6%) were recorded in medical facilities. This indicates that nonhospital deaths have minimal impact on overall trends. Factors such as socioeconomic status, health care accessibility, and comorbidities could affect these findings. Furthermore, the CDC WONDER data set lacks clinical metrics like hemodynamic parameters, inotropic support, or organ failure scores. As a result, the differences in mortality rates based on sex and race may partially mirror unmeasured variations in the severity of shock upon presentation. In contrast to prior studies, this research focuses on adults aged 25 years and older from various racial backgrounds, providing a detailed analysis of mortality trends linked to cardiac arrest in a diverse group. Spanning from 1999 to 2023, our investigation offers a long-term view that enhances the understanding of shifts in cardiac arrest mortality over the past 2 decades.

## Conclusion

Our analysis revealed significant demographic and geographic differences in mortality rates for patients experiencing CS following AMI. Although overall mortality rates have typically decreased, recent data from 2011 to 2021 indicate a concerning rise in mortality trends. This trend emphasizes the need for ongoing monitoring to determine if it will persist or decline. Targeted interventions and improved access to health care are essential to address these disparities and improve patient outcomes within this group.
