# Supplementary material for: Trends in Mortality Among Adults With Acute Myocardial Infarction With Cardiogenic Shock in the United States, 1999-2023
Source: J Soc Cardiovasc Angiogr Interv. 2025 Jun 12;4(7):103711. doi: 10.1016/j.jscai.2025.103711 (PMC12418409; doi:10.1016/j.jscai.2025.103711)
Supplement: Supplemental Tables [file mmc1.docx]

**Supplemental Table S1: Cardiogenic-related mortalities in Adults with Acute Myocardial Infarction, Stratified by Sex and Race in the United States, 1999 to 2023**

| **Deaths** | | | | | | | | |
| --- | --- | --- | --- | --- | --- | --- | --- | --- |
| **Year** | **Overall** | **Women** | **Men** | **NH White** | **NH Black** | **NH Others** | **Hispanic** | **Population** |
| **1999** | 9,507 | 4,577 | 4,930 | 8,192 | 655 | 218 | 422 | 180,408,769 |
| **2000** | 8,659 | 4,142 | 4,517 | 7,435 | 563 | 239 | 408 | 181,984,640 |
| **2001** | 7,880 | 3,634 | 4,246 | 6,704 | 553 | 193 | 412 | 184,305,128 |
| **2002** | 7,539 | 3,561 | 3,978 | 6,362 | 546 | 221 | 393 | 186,208,028 |
| **2003** | 6,969 | 3,188 | 3,781 | 5,852 | 496 | 206 | 407 | 188,090,429 |
| **2004** | 6,526 | 3,039 | 3,487 | 5,446 | 429 | 218 | 425 | 190,205,384 |
| **2005** | 6,250 | 2,897 | 3,353 | 5,117 | 512 | 194 | 422 | 192,551,384 |
| **2006** | 6,085 | 2,848 | 3,237 | 4,965 | 467 | 228 | 418 | 195,019,359 |
| **2007** | 5,999 | 2,770 | 3,229 | 4,909 | 485 | 212 | 389 | 197,403,777 |
| **2008** | 6,037 | 2,686 | 3,351 | 4,925 | 450 | 227 | 427 | 199,795,090 |
| **2009** | 5,702 | 2,503 | 3,199 | 4,682 | 444 | 201 | 372 | 202,107,016 |
| **2010** | 5,991 | 2,658 | 3,333 | 4,819 | 479 | 241 | 440 | 203,891,983 |
| **2011** | 6,057 | 2,648 | 3,409 | 4,906 | 455 | 258 | 433 | 206,592,936 |
| **2012** | 6,318 | 2,676 | 3,642 | 4,973 | 548 | 284 | 500 | 208,826,037 |
| **2013** | 6,397 | 2,665 | 3,732 | 4,998 | 538 | 291 | 558 | 211,085,314 |
| **2014** | 6,810 | 2,869 | 3,941 | 5,344 | 574 | 298 | 577 | 213,809,280 |
| **2015** | 7,341 | 2,948 | 4,393 | 5,591 | 665 | 356 | 688 | 216,553,817 |
| **2016** | 7,910 | 3,151 | 4,759 | 6,115 | 692 | 400 | 673 | 218,641,417 |
| **2017** | 8,240 | 3,249 | 4,991 | 6,235 | 757 | 455 | 775 | 221,447,331 |
| **2018** | 8,603 | 3,367 | 5,236 | 6,403 | 853 | 475 | 848 | 223,311,190 |
| **2019** | 9,005 | 3,635 | 5,370 | 6,740 | 857 | 474 | 916 | 224,981,167 |
| **2020** | 9,209 | 3,593 | 5,616 | 6,722 | 918 | 554 | 1,003 | 226,635,013 |
| **2021** | 10,166 | 3,953 | 6,213 | 7,450 | 985 | 565 | 1,091 | 228,238,412 |
| **2022** | 9,457 | 3,707 | 5,750 | 6,950 | 898 | 556 | 984 | 229,508,599 |
| **2023** | 9,181 | 3,590 | 5,591 | 6,737 | 876 | 524 | 965 | 229,508,599 |
| **Total** | **187,838** | **80,554** | **107,284** | **148,572** | **15,695** | **8,088** | **14,946** | **5,161,110,099** |

**Supplemental Table S2: Cardiogenic Shock related Mortality, Stratified by Place of Death, in Adults with Acute Myocardial Infarction in the United States, 1999 to 2023.**

| **Deaths** | | | | |
| --- | --- | --- | --- | --- |
| **Year** | **Medical Facility** | **Nursing Home/Long-term Care Facility** | **Hospice Facility** | **Home** |
| **1999** | 9,156 | 221 | - | 110 |
| **2000** | 8,371 | 162 | - | 85 |
| **2001** | 7,647 | 132 | - | 73 |
| **2002** | 7,292 | 126 | - | 82 |
| **2003** | 6,695 | 134 | - | 77 |
| **2004** | 6,259 | 129 | - | 72 |
| **2005** | 6,021 | 105 | - | 69 |
| **2006** | 5,835 | 135 | - | 79 |
| **2007** | 5,769 | 105 | 23 | 78 |
| **2008** | 5,755 | 96 | 12 | 64 |
| **2009** | 5,353 | 74 | 35 | 56 |
| **2010** | 5,779 | 86 | 40 | 62 |
| **2011** | 5,835 | 80 | 39 | 72 |
| **2012** | 6,092 | 76 | 49 | 66 |
| **2013** | 6,181 | 66 | 48 | 73 |
| **2014** | 6,605 | 55 | 52 | 73 |
| **2015** | 7,105 | 67 | 73 | 72 |
| **2016** | 7,659 | 53 | 88 | 91 |
| **2017** | 8,020 | 55 | 67 | 70 |
| **2018** | 8,361 | 44 | 102 | 72 |
| **2019** | 8,749 | 65 | 105 | 63 |
| **2020** | 8,943 | 35 | 97 | 94 |
| **2021** | 9,877 | 49 | 98 | 85 |
| **2022** | 9,153 | 51 | 102 | 109 |
| **2023** | 8,894 | 36 | 100 | 107 |
| **Total** | **181,406** | **2,237** | **1,130** | **1,954** |

**Supplemental Table S3: Annual percent change (APC) of cardiogenic-related Age-Adjusted Mortality Rates per 100,000 in Adults with Acute Myocardial Infarction in the United States, 1999 to 2023.**

| **Year Interval** | **APC (95% CI)** | **P-value** |  |
| --- | --- | --- | --- |
| **Overall** | | |  |
| 1999-2004 | -8.94* (-10.82 to -7.86) | < 0.001 |  |
| 2004-2011 | -3.31* (-4.93 to -1.55) | 0.007199 |  |
| 2011-2021 | 3.32* (2.79 to 4.42) | < 0.001 |  |
| 2021-2023 | -5.45* (-8.82 to -1.34) | 0.013997 |  |
| **Men** | | |  |
| 1999-2005 | -8.38* (-9.54 to -7.61) | < 0.001 |  |
| 2005-2011 | -2.24* (-4.17 to -0.30) | 0.027994 |  |
| 2011-2021 | 3.47* (2.98 to 4.40) | < 0.001 |  |
| 2021-2023 | -5.39* (-8.17 to -1.48) | 0.006399 |  |
| **Women** | | |  |
| 1999-2003 | -10.12* (-12.51 to -8.72) | < 0.001 |  |
| 2003-2010 | -4.82* (-6.26 to -2.97) | 0.0012 |  |
| 2010-2021 | 2.29* (1.77 to 3.96) | 0.009998 |  |
| 2021-2023 | -4.95 (-8.51 to 0.45) | 0.076385 |  |
| **NH White** | | |  |
| 1999-2005 | -8.70* (-9.87 to -7.93) | < 0.001 |  |
| 2005-2012 | -2.10* (-3.71 to -0.59) | 0.011598 |  |
| 2012-2021 | 3.28* (2.71 to 4.77) | 0.004399 |  |
| 2021-2023 | -3.91 (-7.05 to 0.20) | 0.064787 |  |
| **NH Black** | | |  |
| 1999-2010 | -4.88* (-6.63 to -3.21) | 0.019196 |  |
| 2010-2021 | 4.24 (-2.86 to 9.06) | 0.064387 |  |
| 2021-2023 | -6.36 (-13.21 to 3.04) | 0.191562 |  |
| **NH Others** | | |  |
| 1999-2009 | -5.93* (-8.53 to -3.33) | 0.024395 |  |
| 2009-2021 | 2.87 (-8.64 to 8.39) | 0.194761 |  |
| 2021-2023 | -2.47 (-6.05 to 2.41) | 0.635073 |  |
| **Hispanic** | | |  |
| 1999-2011 | -4.75* (-5.79 to -3.88) | < 0.001 |  |
| 2011-2021 | 3.94* (3.26 to 5.09) | < 0.001 |  |
| 2021-2023 | -7.77* (-11.89 to -2.45) | 0.0016 |  |
| **Rural areas** | | |  |
| 1999-2008 | -7.55* (-9.31 to -6.41) | < 0.001 |  |
| 2008-2020 | 2.73* (1.67 to 4.19) | < 0.001 |  |
| **Urban areas** | | |  |
| 1999-2008 | -7.14* (-8.25 to -6.41) | < 0.001 |  |
| 2008-2020 | 2.08* (1.40 to 3.05) | < 0.001 |  |
| **Northeast region** | | |  |
| 1999-2005 | -7.78* (-11.83 to -5.45) | 0.003199 |  |
| 2005-2012 | -3.51 (-9.08 to 0.79) | 0.087982 |  |
| 2012-2021 | 2.51 (-4.22 to 8.98) | 0.137972 |  |
| 2021-2023 | -4.47 (-12.18 to 2.06) | 0.289942 |  |
| **South region** | | |  |
| 1999-2009 | -7.24* (-8.35 to -6.42) | < 0.001 |  |
| 2009-2021 | 3.56* (2.82 to 5.09) | 0.011998 |  |
| 2021-2023 | -7.57* (-12.60 to -0.11) | 0.047191 |  |
| **Midwest region** | | |  |
| 1999-2005 | -9.80* (-11.23 to -8.85) | < 0.001 |  |
| 2005-2014 | -0.49 (-1.89 to 0.65) | 0.261548 |  |
| 2014-2021 | 4.12* (3.13 to 6.93) | < 0.001 |  |
| 2021-2023 | -6.83* (-10.68 to -2.09) | 0.002 |  |
| **West region** | | |  |
| 1999-2003 | -8.33* (-11.81 to -6.35) | < 0.001 |  |
| 2003-2012 | -2.77* (-4.01 to -0.65) | 0.029594 |  |
| 2012-2021 | 4.04 (-3.79 to 7.30) | 0.069186 |  |
| 2021-2023 | -2.81 (-6.57 to 3.09) | 0.403519 |  |
| APC = annual percent change; NH = non-Hispanic; * Indicates that the annual percentage change (APC) is significantly different from zero at α = 0.05. AAMR = age-adjusted mortality rate. The data for urbanization is only available till 2020 in the CDC Database. | | |  |
|  |  |  |  |

**Supplemental Table S4: Overall and Sex‐Stratified Cardiogenic Shock Related Age-Adjusted Mortality Rates per 100,000 in Adults with Acute Myocardial Infarction in the United States, 1999 to 2023.**

| **Age-Adjusted Rate (95% CI)** | | | |
| --- | --- | --- | --- |
| **Year** | **Men** | **Women** | **Overall** |
| **1999** | 6.9 (6.7 - 7.1) | 4.3 (4.2 - 4.4) | 5.4 (5.3 - 5.5) |
| **2000** | 6.3 (6.1 - 6.5) | 3.9 (3.7 - 4.0) | 4.8 (4.7 - 4.9) |
| **2001** | 5.8 (5.6 - 6.0) | 3.3 (3.2 - 3.4) | 4.3 (4.2 - 4.4) |
| **2002** | 5.3 (5.2 - 5.5) | 3.2 (3.1 - 3.3) | 4.1 (4.0 - 4.2) |
| **2003** | 4.9 (4.8 - 5.1) | 2.8 (2.7 - 2.9) | 3.7 (3.6 - 3.8) |
| **2004** | 4.4 (4.3 - 4.6) | 2.7 (2.6 - 2.8) | 3.4 (3.3 - 3.5) |
| **2005** | 4.2 (4.0 - 4.3) | 2.5 (2.4 - 2.6) | 3.2 (3.1 - 3.3) |
| **2006** | 3.9 (3.8 - 4.1) | 2.4 (2.3 - 2.5) | 3.1 (3.0 - 3.2) |
| **2007** | 3.8 (3.7 - 4.0) | 2.3 (2.2 - 2.4) | 3.0 (2.9 - 3.0) |
| **2008** | 3.9 (3.7 - 4.0) | 2.2 (2.1 - 2.3) | 2.9 (2.8 - 3.0) |
| **2009** | 3.6 (3.5 - 3.7) | 2.0 (2.0 - 2.1) | 2.7 (2.6 - 2.8) |
| **2010** | 3.7 (3.6 - 3.8) | 2.1 (2.0 - 2.2) | 2.8 (2.7 - 2.9) |
| **2011** | 3.6 (3.5 - 3.7) | 2.1 (2.0 - 2.2) | 2.7 (2.7 - 2.8) |
| **2012** | 3.8 (3.6 - 3.9) | 2.1 (2.0 - 2.1) | 2.8 (2.7 - 2.9) |
| **2013** | 3.7 (3.6 - 3.8) | 2.0 (1.9 - 2.1) | 2.7 (2.7 - 2.8) |
| **2014** | 3.8 (3.7 - 3.9) | 2.1 (2.0 - 2.2) | 2.9 (2.8 - 2.9) |
| **2015** | 4.1 (4.0 - 4.2) | 2.2 (2.1 - 2.2) | 3.0 (3.0 - 3.1) |
| **2016** | 4.4 (4.2 - 4.5) | 2.3 (2.2 - 2.4) | 3.2 (3.1 - 3.2) |
| **2017** | 4.4 (4.3 - 4.5) | 2.3 (2.2 - 2.4) | 3.2 (3.2 - 3.3) |
| **2018** | 4.5 (4.4 - 4.6) | 2.3 (2.2 - 2.4) | 3.3 (3.3 - 3.4) |
| **2019** | 4.5 (4.4 - 4.7) | 2.4 (2.4 - 2.5) | 3.4 (3.3 - 3.5) |
| **2020** | 4.7 (4.5 - 4.8) | 2.4 (2.3 - 2.5) | 3.4 (3.3 - 3.5) |
| **2021** | 5.2 (5.0 - 5.3) | 2.7 (2.6 - 2.7) | 3.8 (3.7 - 3.9) |
| **2022** | 4.7 (4.5 - 4.8) | 2.4 (2.3 - 2.5) | 3.4 (3.3 - 3.5) |
| **2023** | 4.5 (4.4 - 4.7) | 2.3 (2.3 - 2.4) | 3.3 (3.3 - 3.4) |
| **Total** | **4.5 (4.4 - 4.6)** | **2.5 (2.4 - 2.6)** | **3.4 (3.3 - 3.5)** |

**Supplemental Table S5: Race‐Stratified Cardiogenic Shock Related Age-Adjusted Mortality Rates per 100,000 in Adults with Acute Myocardial Infarction in the United States, 1999 to 2023.**

| **Age-Adjusted Rate (95% CI)** | | | | |
| --- | --- | --- | --- | --- |
| **Year** | **NH White** | **NH Black** | **NH Others** | **Hispanic** |
| **1999** | 5.5 (5.4 - 5.6) | 4.4 (4.1 - 4.8) | 4.6 (3.9 - 5.2) | 5.0 (4.5 - 5.5) |
| **2000** | 5.0 (4.8 - 5.1) | 3.8 (3.5 - 4.1) | 4.7 (4.1 - 5.4) | 4.6 (4.1 - 5.0) |
| **2001** | 4.4 (4.3 - 4.5) | 3.6 (3.3 - 3.9) | 3.7 (3.2 - 4.2) | 4.3 (3.9 - 4.8) |
| **2002** | 4.1 (4.0 - 4.2) | 3.5 (3.2 - 3.8) | 3.9 (3.4 - 4.5) | 3.9 (3.5 - 4.2) |
| **2003** | 3.8 (3.7 - 3.9) | 3.1 (2.8 - 3.4) | 3.5 (3.0 - 4.0) | 3.8 (3.4 - 4.2) |
| **2004** | 3.5 (3.4 - 3.6) | 2.6 (2.4 - 2.9) | 3.4 (2.9 - 3.9) | 3.8 (3.4 - 4.1) |
| **2005** | 3.2 (3.1 - 3.3) | 3.1 (2.8 - 3.4) | 2.9 (2.4 - 3.3) | 3.6 (3.3 - 4.0) |
| **2006** | 3.1 (3.0 - 3.2) | 2.7 (2.5 - 3.0) | 3.1 (2.6 - 3.5) | 3.4 (3.1 - 3.8) |
| **2007** | 3.0 (2.9 - 3.1) | 2.8 (2.5 - 3.0) | 2.8 (2.4 - 3.1) | 3.0 (2.6 - 3.3) |
| **2008** | 3.0 (2.9 - 3.0) | 2.5 (2.3 - 2.7) | 2.8 (2.5 - 3.2) | 3.1 (2.8 - 3.4) |
| **2009** | 2.8 (2.7 - 2.9) | 2.4 (2.2 - 2.6) | 2.3 (2.0 - 2.6) | 2.6 (2.3 - 2.8) |
| **2010** | 2.8 (2.7 - 2.9) | 2.5 (2.3 - 2.7) | 2.8 (2.4 - 3.2) | 2.9 (2.6 - 3.2) |
| **2011** | 2.8 (2.7 - 2.9) | 2.2 (2.0 - 2.4) | 2.8 (2.4 - 3.1) | 2.6 (2.3 - 2.8) |
| **2012** | 2.8 (2.7 - 2.9) | 2.7 (2.4 - 2.9) | 2.8 (2.5 - 3.1) | 2.8 (2.6 - 3.1) |
| **2013** | 2.7 (2.7 - 2.8) | 2.4 (2.2 - 2.6) | 2.7 (2.4 - 3.0) | 3.0 (2.8 - 3.3) |
| **2014** | 2.9 (2.8 - 3.0) | 2.6 (2.4 - 2.8) | 2.6 (2.3 - 2.9) | 3.0 (2.7 - 3.2) |
| **2015** | 3.0 (2.9 - 3.1) | 2.8 (2.6 - 3.1) | 2.9 (2.6 - 3.2) | 3.2 (2.9 - 3.4) |
| **2016** | 3.2 (3.2 - 3.3) | 2.9 (2.7 - 3.1) | 3.1 (2.8 - 3.4) | 3.0 (2.8 - 3.2) |
| **2017** | 3.2 (3.1 - 3.3) | 3.0 (2.8 - 3.3) | 3.3 (3.0 - 3.6) | 3.3 (3.1 - 3.5) |
| **2018** | 3.3 (3.2 - 3.4) | 3.3 (3.1 - 3.5) | 3.2 (2.9 - 3.5) | 3.5 (3.2 - 3.7) |
| **2019** | 3.4 (3.3 - 3.5) | 3.3 (3.1 - 3.5) | 3.2 (2.9 - 3.5) | 3.6 (3.4 - 3.9) |
| **2020** | 3.3 (3.3 - 3.4) | 3.4 (3.2 - 3.7) | 3.5 (3.2 - 3.8) | 3.7 (3.5 - 4.0) |
| **2021** | 3.8 (3.7 - 3.9) | 3.7 (3.5 - 4.0) | 3.6 (3.3 - 3.9) | 4.0 (3.7 - 4.2) |
| **2022** | 3.4 (3.3 - 3.5) | 3.3 (3.1 - 3.5) | 3.5 (3.2 - 3.7) | 3.4 (3.2 - 3.6) |
| **2023** | 3.4 (3.3 - 3.4) | 3.2 (3.0 - 3.5) | 3.3 (3.0 - 3.5) | 3.4 (3.2 - 3.7) |
| **Total** | **3.4 (3.3 - 3.5)** | **3.0 (2.8 - 3.3)** | **3.2 (2.9 - 3.6)** | **3.5 (3.2 - 3.8)** |
| NH = non-Hispanic. | | | | |

**Supplemental Table S6: Cardiogenic Shock related Age-Adjusted Mortality Rates per 100,000, Stratified by States, in Adults with Acute Myocardial Infarction in the United States, 1999 to 2023.**

| **State** | **Age-Adjusted Rate (95% CI)** |
| --- | --- |
| Alabama | 3.7 (3.6 - 3.9) |
| Alaska | 2.4 (2.0 - 2.8) |
| Arizona | 3.2 (3.1 - 3.4) |
| Arkansas | 4.2 (4.0 - 4.4) |
| California | 3.5 (3.4 - 3.5) |
| Colorado | 2.4 (2.3 - 2.5) |
| Connecticut | 3.1 (3.0 - 3.2) |
| Delaware | 3.2 (2.9 - 3.5) |
| District of Columbia | 3.0 (2.6 - 3.3) |
| Florida | 3.0 (2.9 - 3.1) |
| Georgia | 3.4 (3.3 - 3.5) |
| Hawaii | 3.8 (3.6 - 4.1) |
| Idaho | 2.7 (2.5 - 3.0) |
| Illinois | 2.5 (2.4 - 2.6) |
| Indiana | 4.2 (4.1 - 4.4) |
| Iowa | 3.3 (3.2 - 3.5) |
| Kansas | 3.2 (3.0 - 3.3) |
| Kentucky | 4.3 (4.2 - 4.5) |
| Louisiana | 3.2 (3.0 - 3.3) |
| Maine | 3.8 (3.6 - 4.1) |
| Maryland | 2.6 (2.4 - 2.7) |
| Massachusetts | 2.9 (2.8 - 3.0) |
| Michigan | 3.6 (3.5 - 3.7) |
| Minnesota | 2.3 (2.2 - 2.4) |
| Mississippi | 3.8 (3.6 - 3.9) |
| Missouri | 3.3 (3.2 - 3.5) |
| Montana | 2.5 (2.3 - 2.8) |
| Nebraska | 3.1 (2.9 - 3.3) |
| Nevada | 3.7 (3.5 - 3.9) |
| New Hampshire | 3.2 (2.9 - 3.4) |
| New Jersey | 3.1 (3.0 - 3.2) |
| New Mexico | 2.7 (2.5 - 2.8) |
| New York | 2.7 (2.6 - 2.7) |
| North Carolina | 3.4 (3.3 - 3.5) |
| North Dakota | 4.2 (3.8 - 4.6) |
| Ohio | 3.3 (3.3 - 3.4) |
| Oklahoma | 3.7 (3.5 - 3.8) |
| Oregon | 2.8 (2.6 - 2.9) |
| Pennsylvania | 4.0 (3.9 - 4.0) |
| Rhode Island | 4.3 (4.0 - 4.6) |
| South Carolina | 4.1 (4.0 - 4.3) |
| South Dakota | 3.2 (2.9 - 3.5) |
| Tennessee | 4.2 (4.1 - 4.4) |
| Texas | 4.0 (3.9 - 4.0) |
| Utah | 2.7 (2.5 - 2.9) |
| Vermont | 2.9 (2.6 - 3.2) |
| Virginia | 2.9 (2.8 - 2.9) |
| Washington | 3.6 (3.5 - 3.7) |
| West Virginia | 5.3 (5.0 - 5.5) |
| Wisconsin | 2.4 (2.3 - 2.5) |
| Wyoming | 2.6 (2.3 - 3.0) |

**Supplemental Table S7: Cardiogenic Shock related Age-Adjusted Mortality Rates per 100,000, Stratified by Census Region, in Adults with Acute Myocardial Infarction in the United States, 1999 to 2023.**

|  | **Census Region: NorthEast** | **Census Region: Midwest** | **Census Region:  South** | **Census Region:  West** |
| --- | --- | --- | --- | --- |
| **Year** | **Age-Adjusted Rate (95% CI)** | **Age-Adjusted Rate (95% CI)** | **Age-Adjusted Rate (95% CI)** | **Age-Adjusted Rate (95% CI)** |
| **1999** | 5.2 (4.9 - 5.4) | 5.2 (5.0 - 5.4) | 5.9 (5.7 - 6.1) | 4.9 (4.7 - 5.1) |
| **2000** | 5.0 (4.7 - 5.2) | 4.6 (4.4 - 4.8) | 5.1 (5.0 - 5.3) | 4.5 (4.2 - 4.7) |
| **2001** | 4.5 (4.3 - 4.7) | 4.2 (4.0 - 4.4) | 4.5 (4.3 - 4.7) | 3.9 (3.7 - 4.1) |
| **2002** | 4.0 (3.8 - 4.2) | 3.8 (3.6 - 3.9) | 4.5 (4.3 - 4.7) | 3.8 (3.6 - 4.0) |
| **2003** | 4.0 (3.8 - 4.2) | 3.3 (3.1 - 3.5) | 3.9 (3.8 - 4.1) | 3.5 (3.4 - 3.7) |
| **2004** | 3.4 (3.2 - 3.6) | 3.1 (3.0 - 3.3) | 3.6 (3.4 - 3.7) | 3.4 (3.3 - 3.6) |
| **2005** | 3.3 (3.1 - 3.4) | 2.9 (2.7 - 3.0) | 3.3 (3.2 - 3.5) | 3.3 (3.1 - 3.5) |
| **2006** | 3.1 (2.9 - 3.3) | 2.8 (2.6 - 2.9) | 3.3 (3.2 - 3.4) | 3.0 (2.8 - 3.1) |
| **2007** | 3.0 (2.8 - 3.1) | 2.7 (2.6 - 2.9) | 3.1 (2.9 - 3.2) | 3.1 (2.9 - 3.2) |
| **2008** | 3.0 (2.9 - 3.2) | 2.7 (2.5 - 2.8) | 3.0 (2.9 - 3.2) | 2.9 (2.7 - 3.0) |
| **2009** | 2.7 (2.5 - 2.8) | 2.6 (2.5 - 2.7) | 2.7 (2.6 - 2.8) | 2.8 (2.6 - 2.9) |
| **2010** | 2.7 (2.5 - 2.8) | 2.7 (2.6 - 2.9) | 2.9 (2.8 - 3.0) | 2.9 (2.7 - 3.0) |
| **2011** | 2.7 (2.5 - 2.8) | 2.7 (2.6 - 2.9) | 2.8 (2.7 - 2.9) | 2.8 (2.6 - 2.9) |
| **2012** | 2.5 (2.4 - 2.7) | 2.8 (2.6 - 2.9) | 2.9 (2.8 - 3.0) | 2.8 (2.7 - 3.0) |
| **2013** | 2.6 (2.5 - 2.8) | 2.6 (2.4 - 2.7) | 2.9 (2.8 - 3.0) | 2.7 (2.6 - 2.9) |
| **2014** | 2.7 (2.6 - 2.9) | 2.7 (2.5 - 2.8) | 3.1 (3.0 - 3.2) | 2.8 (2.7 - 3.0) |
| **2015** | 2.8 (2.7 - 3.0) | 2.6 (2.5 - 2.8) | 3.3 (3.2 - 3.4) | 3.1 (3.0 - 3.3) |
| **2016** | 2.9 (2.7 - 3.0) | 3.0 (2.8 - 3.1) | 3.4 (3.3 - 3.6) | 3.2 (3.0 - 3.3) |
| **2017** | 2.7 (2.6 - 2.9) | 2.9 (2.7 - 3.0) | 3.6 (3.5 - 3.8) | 3.3 (3.1 - 3.4) |
| **2018** | 2.9 (2.7 - 3.1) | 3.2 (3.0 - 3.4) | 3.6 (3.5 - 3.7) | 3.2 (3.1 - 3.4) |
| **2019** | 2.9 (2.7 - 3.1) | 3.4 (3.2 - 3.5) | 3.7 (3.6 - 3.8) | 3.4 (3.2 - 3.5) |
| **2020** | 2.9 (2.8 - 3.1) | 3.3 (3.1 - 3.4) | 3.7 (3.6 - 3.8) | 3.4 (3.3 - 3.6) |
| **2021** | 3.4 (3.2 - 3.5) | 3.5 (3.3 - 3.7) | 4.1 (3.9 - 4.2) | 4.1 (3.9 - 4.2) |
| **2022** | 3.0 (2.8 - 3.1) | 3.2 (3.1 - 3.4) | 3.5 (3.4 - 3.6) | 3.7 (3.6 - 3.9) |
| **2023** | 2.9 (2.7 - 3.0) | 3.1 (3.0 - 3.3) | 3.5 (3.4 - 3.6) | 3.6 (3.4 - 3.7) |
| **Total** | **3.2 (3.1 - 3.4)** | **3.2 (3.0 - 3.3)** | **3.6 (3.5 - 3.7)** | **3.4 (3.2 - 3.5)** |

**Supplemental Table S8: Cardiogenic Shock-Related Age-Adjusted Mortality Rates per 100,000, Stratified by Urban-Rural Classification, in Adults with Acute Myocardial Infarction in the United States, 1999 to 2020.**

| **Age-Adjusted Rate (95% CI)** | | |  |
| --- | --- | --- | --- |
| **Year** | **Urban** | **Rural** |  |
| **1999** | 5.1 (5.0 - 5.2) | 6.5 (6.2 - 6.8) |  |
| **2000** | 4.6 (4.5 - 4.7) | 5.7 (5.4 - 5.9) |  |
| **2001** | 4.2 (4.1 - 4.3) | 5.2 (4.9 - 5.4) |  |
| **2002** | 3.9 (3.8 - 4.0) | 5.1 (4.8 - 5.3) |  |
| **2003** | 3.6 (3.5 - 3.7) | 4.4 (4.2 - 4.6) |  |
| **2004** | 3.3 (3.2 - 3.4) | 4.0 (3.8 - 4.2) |  |
| **2005** | 3.1 (3.0 - 3.2) | 3.7 (3.5 - 3.9) |  |
| **2006** | 3.0 (2.9 - 3.1) | 3.5 (3.3 - 3.7) |  |
| **2007** | 2.8 (2.8 - 2.9) | 3.6 (3.4 - 3.8) |  |
| **2008** | 2.8 (2.7 - 2.9) | 3.5 (3.3 - 3.7) |  |
| **2009** | 2.7 (2.6 - 2.7) | 3.0 (2.8 - 3.2) |  |
| **2010** | 2.7 (2.6 - 2.8) | 3.4 (3.2 - 3.5) |  |
| **2011** | 2.6 (2.5 - 2.7) | 3.4 (3.2 - 3.5) |  |
| **2012** | 2.6 (2.6 - 2.7) | 3.5 (3.3 - 3.7) |  |
| **2013** | 2.7 (2.6 - 2.7) | 3.2 (3.1 - 3.4) |  |
| **2014** | 2.8 (2.7 - 2.8) | 3.4 (3.2 - 3.6) |  |
| **2015** | 2.9 (2.8 - 3.0) | 3.6 (3.4 - 3.8) |  |
| **2016** | 3.0 (3.0 - 3.1) | 3.9 (3.7 - 4.1) |  |
| **2017** | 3.1 (3.0 - 3.1) | 4.0 (3.8 - 4.2) |  |
| **2018** | 3.2 (3.1 - 3.3) | 4.0 (3.8 - 4.2) |  |
| **2019** | 3.2 (3.1 - 3.3) | 4.4 (4.2 - 4.6) |  |
| **2020** | 3.3 (3.2 - 3.3) | 4.2 (4.0 - 4.3) |  |
| **Total** | **3.2 (3.1 - 3.2)** | **4.0 (4.0 - 4.0)** |  |
| The data for urbanization is only available till 2020 in the CDC Database | | |  |
|  |  |  |  |
